# Exploring the interaction dynamics of eukaryotic translation initiation factor 2

**DOI:** 10.1042/BST20253022

**Published:** 2025-05-23

**Authors:** Assen Marintchev

**Affiliations:** 1 Department of Pharmacology, Physiology, & Biophysics, Boston University Chobanian & Avedisian School of Medicine, Boston, MA, U.S.A.

**Keywords:** eIF2, ISR, translation initiation, translation pre-initiation complex, translation regulation

## Abstract

Eukaryotic translation initiation typically involves recruitment of the 43S ribosomal pre-initiation complex (PIC) to the 5′-end of the mRNA to form the 48S PIC, followed by scanning in search of a start codon in a favorable nucleotide complex. The start codon is recognized through base-pairing with the anticodon of the initiator Met-tRNA_i_. The stringency of start codon selection controls the probability of initiation from a start codon in a suboptimal nucleotide context. Met-tRNA_i_ itself is recruited to the 43S PIC by the eukaryotic translation initiation factor 2 (eIF2), in the form of the eIF2-GTP•Met-tRNA_i_ ternary complex (TC). GTP hydrolysis by eIF2, promoted by its GTPase-activating protein eIF5, leads to the release of eIF2-GDP from the PIC. Recycling of eIF2-GDP to TC is promoted by the guanine nucleotide exchange factor eIF2B. Its inhibition by a number of stress factors triggers the integrated stress response (ISR). This review describes the recent advances in elucidating the interactions of eIF2 and its partners, with an emphasis on the timing and dynamics of their binding to, and release from the PIC. Special attention is given to the regulation of the stringency of start codon selection and the ISR. The discussion is mostly limited to translation initiation in mammals and budding yeast.

Eukaryotic translation initiation is a complex multistep process or rather a set of parallel and interrelated alternative processes. The focus of this review is eukaryotic translation initiation factor 2 (eIF2), and due to space considerations, we will limit our discussion to the ‘canonical’ translation initiation process, where eIF2 is the protein that brings the initiator Met-tRNA_i_ to the 43S ribosomal pre-initiation complex (PIC); eIF4E (as part of eIF4F) is the relevant cap-binding protein; and the 43S PIC is recruited to the 5′-end of the mRNA by eIF4F to form the 48S PIC, which then scans in search of a start codon in the proper sequence context (aka Kozak sequence) (reviewed in [1–4]). In this review, 43S PIC refers to the complex of the small (40S) ribosomal subunit with eIFs 1, 1A, 2, 3, and 5, and the initiator Met-tRNA_i_, whereas 48S PIC refers to a 43S PIC bound to the mRNA, either scanning or already located at the start codon, to reflect commonly used nomenclature (see e.g., [4]). In alternative nomenclatures, the scanning 48S PIC has been referred to as scanning 43S PIC, and the 48S PIC that has reached the start codon – as 48S initiation complex (IC) (see e.g., [5]).

Even though the process of translation initiation has been studied for decades, many of its key aspects are still not fully understood or a subject of controversy. For example, it is not clear whether in mammals, the 48S PIC-bound eIF4F remains also bound to the 5′-cap (‘tethered’ model, see e.g., [6]) or lets go of it (‘untethered’ model, see e.g., [7,8]) as in *Saccharomyces cerevisiae* [9,10]. Likewise, while it is commonly thought that the 43S PIC binds to the mRNA downstream from the cap-binding complex, leaving it unable to inspect the first 15–30 nucleotides for the presence of a start codon, there are reports that it can inspect the mRNA from the 5′-end, either directly (see e.g., [8]) or by bi-directional scanning (see e.g., [7]). These topics are, however, beyond the scope of this review. The key questions discussed here are the dynamics of interactions involving eIF2 and its binding partners, the stringency of start codon selection, as well as the recycling of the inactive eIF2-GDP back to eIF2-GTP and the eIF2-GTP•Met-tRNA_i_ ternary complex (TC) by its guanine nucleotide exchange factor (GEF) eIF2B and the integrated stress response (ISR) that is triggered when eIF2B activity is inhibited. Special attention will be paid to the timing of binding and release of individual PIC components and which interactions among them are present at which stages of translation initiation, in particular as it relates to eIF2 and its binding partners. I apologize in advance for all articles that were not cited here because they were deemed outside of the scope of this review, due to space considerations, or were inadvertently omitted.

Let us first briefly introduce the major players (aka the translation initiation ‘alphabet soup’) and the main steps in the process. eIF4F is composed of the cap-binding protein eIF4E, the RNA helicase eIF4A, and the scaffold protein eIF4G. eIF4G stimulates the eIF4A helicase activity, along with eIFs 4B and 4H and also interacts with the polyA-binding protein (PABP). After eIF4F recruits the 43S PIC to the mRNA, it remains associated with the scanning 48S PIC. The 43S PIC itself is composed of the small (40S) ribosomal subunit, eIF1, eIF1A, eIF2, Met-tRNA_i_, eIF3, and eIF5. eIF3 is a large protein complex with multiple roles in translation, ranging from 5 to 6 subunits in *S. cerevisiae* to 13 subunits in mammals. Most of eIF3 is located on the ‘back’, solvent-exposed surface of the 40S, away for the interface with the large (60S) ribosomal subunit, and, along with eIF4F, it remains transiently associated with the ribosome after the start of translation elongation [6,11,12]. eIF2 recruits Met-tRNA_i_, in the form of the eIF2-GTP•Met-tRNA_i_ TC, to the 43S PIC. eIF2 also interacts with almost all other PIC components. Upon start codon selection, eIF2 hydrolyzes GTP with the help of its GTPase-activating protein (GAP) eIF5 and hands over the Met-tRNA_i_ to a second GTPase, eIF5B, which, together with eIF1A, promotes 60S subunit joining to form the 80S ribosome (Figure 1) (reviewed in [1–4]).

**Figure 1:**
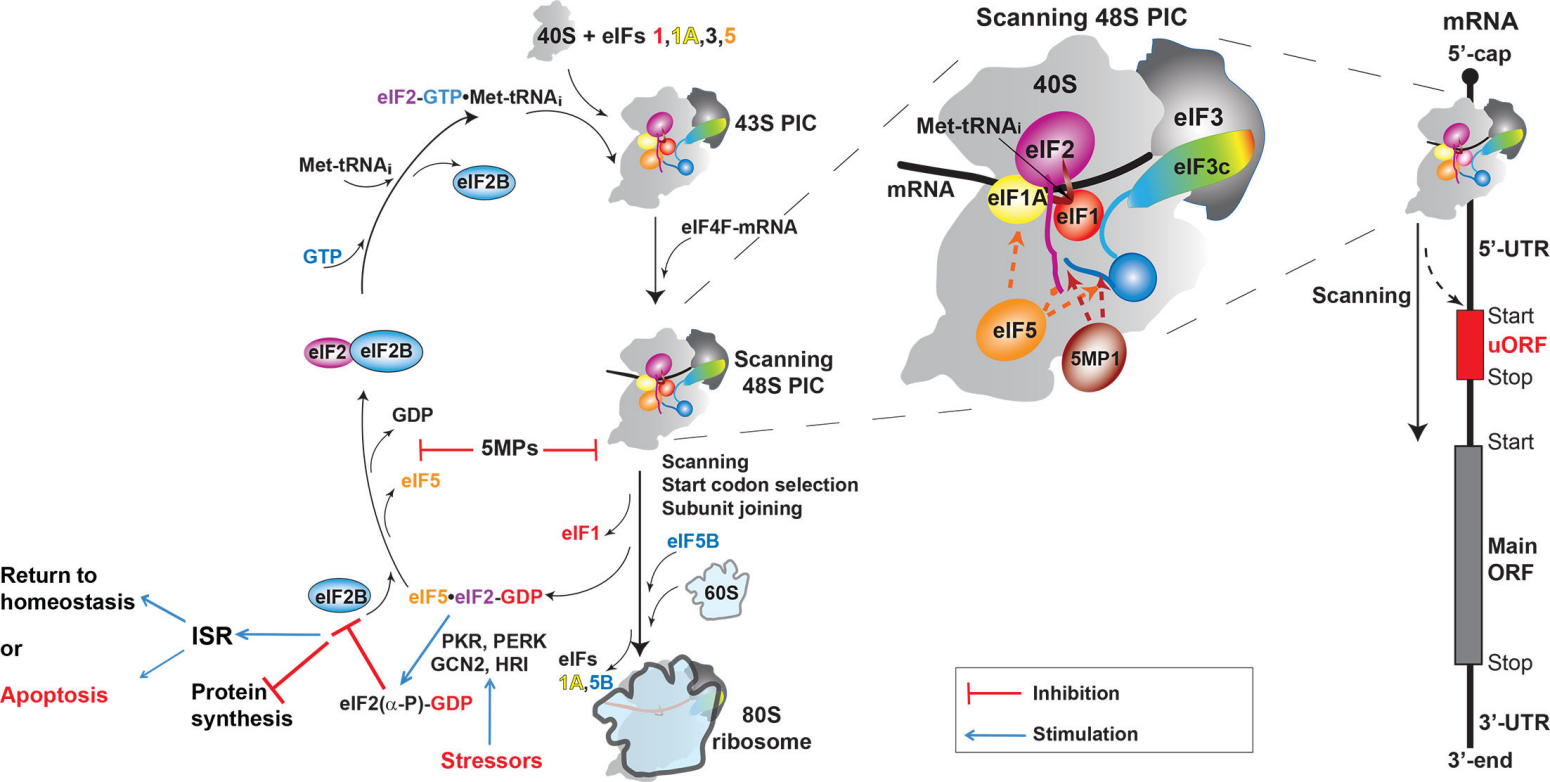
Translation initiation, the pre-initiation complex (PIC), and the integrated stress response (ISR). Folded domains are shown as shapes; intrinsically disordered regions – as lines. eIF3c is colored rainbow from the N-terminus (blue) to C-terminus (red). Some proteins are not shown, including the mRNA cap-binding complex eIF4F (eIF4E/G/A) and parts of eIF3. Interactions of eIF5 and 5MP1 are shown with dashed arrows and color-coded. As discussed in the text, not all of these interactions may be taking place on the scanning 48S PIC. Upon start codon selection, the NTD of eIF5 displaces eIF1 on the 40S ribosomal subunit. For simplicity, eIF5 and eIF2-GDP are shown leaving the 48S PIC together; however, as discussed in the text, they may leave separately and then form a complex off the PIC. PKR, PERK, GCN2, and HRI are stress-activated kinases that phosphorylate eIF2α and induce the ISR. An example of an mRNA with an inhibitory upstream open reading frame (uORF), colored red, with a start codon in a suboptimal context is shown on the right. The ORFs (protein-coding sequences) are shown as rectangles. The fraction of PICs that bypass the uORF to reach the main ORF, colored gray, is regulated by the stringency of start codon selection and the nucleotide context of the start codon. 5′-UTR, 5′-untranslated region; 3′-UTR, 3′-untranslated region; 40S, small ribosomal subunit; 60S, large ribosomal subunit.

eIF2 is a heterotrimeric GTPase, composed of α, β, and γ subunits (called eIF2S1, S2, and S3, respectively, in online databases). The eIF2γ subunit is the actual GTPase, while eIF2α and β have accessory and regulatory functions. Phosphorylation of eIF2α under a number of stress conditions by any one of the stress kinases GCN2, PKR, PERK, and HRI turns eIF2 into an inhibitor of its GEF eIF2B, which triggers the ISR (Figure 1, reviewed in [1,3,4,13]). Because of its role as an ISR marker, eIF2α is arguably the best known eIF and also the most frequently misnamed – it is often called elF2a, but sometimes even elF2A, which is a completely unrelated protein. eIF2, eIF1, eIF3, eIF5, and the Met-tRNA_i_ assemble into the multifactor complex (MFC). Within the MFC, all proteins interact with each other, although the strength of individual interactions varies between mammals and *S. cerevisiae* [14,15]. The interactions of eIF1 with eIF2 and eIF3c have been observed within the PIC [16–20], although only a single helix from eIF3c is observed interacting with eIF1 in PIC structures while at least in *S. cerevisiae*, eIF3c has multiple binding sites for eIF1 [21]. eIF5-CTD binds to eIF2β, eIF3c, and weakly to eIF1 [14,22–26], as well as to eIF1A [24,25,27]. Unfortunately, eIF5-CTD is not visible in Cryo-EM structures of yeast or mammalian 43S or 48S PICs; thus, none of its interactions could be observed, either. The only case where eIF5-CTD was seen was the structure of the *T. cruzi* 43S PIC, where it was observed contacting eIF2 [28].

If the 48S PIC encounters an AUG codon in optimal Kozak sequence context, the Met-tRNA_i_ anticodon base-pairs with the start codon; the 48S PIC converts from an ‘open’, scanning-competent, to a ‘closed’, scanning-incompetent conformation; and translation almost always initiates from that start codon. The probability of initiation at a suboptimal start codon depends on the stringency of start codon selection. If the 48S PIC encounters an AUG in a suboptimal context or a near-cognate start codon (e.g., CUG or GUG) in an optimal context, the 48S PIC may recognize it and adopt a ‘closed’ conformation, but it would be less stable, and the 48S PIC may either initiate at that start codon or revert back to the open state and continue scanning (Figure 1, right). While all PIC components influence the stringency of start codon selection, either directly or indirectly, the central players in this process are eIF1 and eIF5. eIF1 is bound next to the Met-tRNA_i_ in the ribosomal P-site and is displaced from there when the tRNA base-pairs with the start codon. The N-terminal domain (NTD) of eIF5 then takes the place of eIF1 on the 40S subunit. eIF5 also promotes GTP hydrolysis by eIF2, which commits the 48S PIC to initiate at that start codon (reviewed in [1–4]). Higher eIF1 concentrations increase the stringency of start codon selection, whereas higher eIF5 concentrations lower it. In fact, eIF1 and eIF5 regulate their own and each other’s translation by modulating the stringency of start codon selection: the eIF1 start codon is in a suboptimal context, whereas the 5′-untranslated region (5′-UTR) of eIF5 contains inhibitory upstream open reading frames (uORFs) in a suboptimal context. Thus, higher eIF1 concentration decreases translation of its mRNA while increasing translation of eIF5, whereas higher eIF5 concentration has the opposite effect [29–31]. The regulatory eIF5-mimic protein 5MP competes with eIF5 for binding to eIF2 and the PIC (Figure 1) [32–34]. Mammals have two 5MP isoforms: 5MP1 and 5MP2, previously known as basic leucine zipper – W2 domain 2 (BZW2) and BZW1, respectively; but their NTD was predicted by us [35] and AlphaFold [36,37] to be an MA3 HEAT domain homologous to the second HEAT domain of eIF4G, not a leucine zipper. The C-terminal domains (CTD) of eIF5, eIF2Bε, and the 5MPs are W2 HEAT domains homologous to each other and to the third HEAT domain of eIF4G [38]. By competing with eIF5, the 5MPs increase the stringency of start codon selection, similar to eIF1, and their translation efficiency is auto- and cross-regulated with those of eIF1 and eIF5 [34,39]. While the regulation of eIF1 translation is conserved in *S. cerevisiae*, this does not appear to be the case for eIF5. Most *Ascomycetes* (yeast), including *S. cerevisiae,* do not have a 5MP homolog, whereas *Basidiomycetes* (mushrooms) do have a 5MP [40,41].

eIF2-GDP and eIF5 bind to each other with high affinity [15,42–44] and eIF2-GDP may be released from the 48S PIC together with its GAP eIF5, at least in *S. cerevisiae* [43,44]. The GEF eIF2B has to displace eIF5 before it can recycle eIF2-GDP back to eIF2-GTP and eIF2-GTP•Met-tRNA_i_ TC (Figure 1). Thus, eIF5 also plays the role of a GDP-dissociation inhibitor (GDI), and eIF2B also plays the role of a GDI-dissociating factor (GDF) [43–45]. According to the GDI definition used in ref. [44] that a GDI directly slows down GDP release, only *S. cerevisiae* eIF5, but not human eIF5 is a GDI [15,44]. However, the main inhibitory effect of eIF5 in both yeast and mammals is mediated by preventing eIF2B binding to eIF2, not further slowing down the already slow spontaneous exchange rate of free eIF2-GDP. According to the original definition that the GDI interferes with the GEF binding [46], both eIF5′s are GDIs. 5MP1 and 5MP2 compete for eIF2 binding not only with eIF5 but also with eIF2B [32,47]. Thus, like eIF5, they can also act as GDIs.

The CTDs of eIF5, eIF2Bε, and the 5MPs bind to the eIF2β N-terminal tail (eIF2β-NTT). eIF2β-NTT has three lysine repeats (K-boxes) important for the interactions with eIF5, eIF2Bε, and the 5MPs [14,23,32,34]. We recently found that eIF2β-NTT contains three tandem binding sites centered on the three K-boxes. eIF5, eIF2Bε, and 5MP1 can each bind to all three K-boxes with similar but not identical affinities. Three proteins can simultaneously bind to the same eIF2β-NTT, but when they do, they reduce each other’s affinity [47]. This led us to propose that the simultaneous binding and mutual destabilization allow eIF5 and eIF2B to speed up each other’s displacement from eIF2 (as in the GDF function of eIF2B) and mediate the regulatory functions of the 5MPs, which compete with eIF5 and eIF2B for eIF2 and modulate their activities [47].

CK2 is important for cell proliferation and differentiation, has hundreds of substrates, and is constitutively active in most cells under most conditions [48–51], but inactive under certain chronic stress conditions [48,52–54]. In mammals, CK2 phosphorylates eIF5 [49], eIF2Bε [55], and the 5MPs [47] within their eIF2β-binding regions, which increases their affinities for eIF2β-NTT *in vitro* [47,56], stimulates the activities of eIF2B *in vitro* [55], eIF5 in cell culture [49], and likely also the 5MPs. The observation that CK2 phosphorylation stabilizes multiple, often competing interactions, suggests that it remodels the entire system, making it more efficient, although the effects of phosphorylation have not yet been examined in the context of the PIC. This raises the possibility that under chronic stress, when overall translation is suppressed and the concentrations of ribosomes and translation factors are likely proportionally reduced, the stability and even composition of translation complexes may be significantly modulated. Mammalian mTORC1 and CK2 bind to eIF2β and phosphorylate its NTT [48] on the periphery of the binding sites for eIF5, eIF2Bε, and the 5MPs, allowing NCK1 to bind and recruit PP1, which in turn dephosphorylates eIF2α and blunts the ISR when mTORC1 and/or CK2 are active [48].

When (and how stably) are eIF1 and eIF5 bound to the scanning 48S PIC? eIF1 is required for proper scanning and start codon selection. Therefore, it must be present on the scanning 48S PIC, at least most of the time, on most PICs. eIF5, on the other hand, is not required for scanning but is required at the start codon, where it replaces eIF1 at the P-site and promotes GTP hydrolysis by eIF2, which is required for the 48S PIC to commit to initiating at this start codon. On the other hand, the stringency of start codon selection depends on eIF5 concentration; therefore, the eIF5 occupancy at the start codon depends on free eIF5 concentration [30,57,58]. Therefore, eIF5 must be present on every 48S PIC at the start codon, at least transiently to promote initiation at that codon, but not 100% of the time, because then its occupancy would not have been dependent on free eIF5 concentration. eIF5-NTD is first observed in Cryo-EM structures of PICs once it replaces eIF1 upon start codon selection [4,19,59]. The only PIC structure that contains eIF5-CTD (and the only instance when eIF5 was observed on the PIC prior to start codon selection) is the *T. cruzi* 43S PIC structure [28]. This is also the only instance when a putative portion of the intrinsically disordered eIF2β-NTT was observed – as density contacting eIF5-CTD. At least at suboptimal start codons, the release of eIF1 is reversible, and it can compete with eIF5-NTD for binding to the 40S. A recent report using SM-FRET provided direct evidence that mammalian eIF1 and eIF5 binding to the 48S PIC is indeed dynamic and that they compete for the binding site on the 40S upon start codon selection [60]. Remarkably, the authors did not observe eIF5 on the 48S PIC until start codon selection and proposed that in mammals, eIF5 is not present on the scanning 48S PIC [60]. While the failure to observe eIF5 in a given Cryo-EM structure may mean that it is tethered to the PIC via disordered segments and remains invisible, failure to detect it on the scanning 48S PIC using SM-FRET or even with direct excitation is not easy to rationalize. This finding is highly unexpected and difficult to reconcile with many previous reports, raising questions we do not seem to have the answer to. The discussion below relies on the assumption that if an interaction exists *in vitro*, it also occurs *in vivo* on and/or off the PIC, and it has a function, or it would not have been conserved during evolution. (1) eIF5 is a stable component of the MFC, not only in *S. cerevisiae* [14], but also in mammals [15]. Of course, not all interactions within the MFC are retained within the PIC. However, if eIF5 is part of the MFC and falls off when the MFC binds to the 40S, why was it part of the MFC in the first place? At present, there are no known functions of eIF5 within the MFC or of the MFC itself off the PIC. (2) What would prevent eIF5 from interacting dynamically with the eIF2β-NTT, which is not visible in PIC structures and likely remains disordered? A possible explanation is that the authors used recombinant bacterially expressed eIF5, which would not be phosphorylated by CK2 and thus have at least an order of magnitude lower affinity for eIF2β [47]. (3) The interactions of eIF5 with eIFs 1, 1A, and 3c are conserved from fungi to mammals [14,15,24,25] and such ‘late arrival’ of mammalian eIF5 is also difficult to reconcile with the model that eIF1A and eIF3c displace eIF2β-NTT from eIF5-CTD on the PIC [24,25] – if eIF5 first binds when the 48S PIC is already at the start codon, does the incoming eIF5 bind to eIF2β first or to eIF1A/3c first? Furthermore, the eIF1A N-terminus may no longer be accessible in the 48S PIC at the start codon [19]; then, when and where does eIF5-CTD bind to eIF1A? It appears that our understanding of the dynamics of eIF5 binding to the PIC is shifting from eIF5 being always present in the PIC, to coming later, after scanning. Or the truth could be somewhere in between. As discussed earlier, the dependence of the stringency of start codon selection on eIF5 concentration indicates that eIF5 is not present on the PIC 100% of the time; therefore, even if it bound initially to the 43S PIC (whether alone or as part of the MFC), this interaction is likely dynamic, involving rounds of binding and release. eIF5 may bind dynamically (on and off) to the scanning 48S PIC by contacting eIF2β-NTT and likely also eIF3c-NTT. Then, upon start codon selection, eIF5-NTD replaces eIF1 from its binding site on the 40S (and becomes visible in Cryo-EM structures, while eIF5-CTD remains dynamic and invisible). However, this may not be the case under certain chronic stress conditions, where CK2 is inactive, eIF5-CTD becomes dephosphorylated and its binding affinity for eIF2β weakens significantly (to a *Kd* in the μM range [47,56]). Under those conditions, eIF5 may indeed arrive after start codon selection as observed by Grosely and co-authors [60].

When (and how stably) is the eIF2-GTP•Met-tRNA_i_ TC bound to the scanning 48S PIC? For the purposes of this discussion, we focus on PICs, where eIF2 (and not alternative factors) recruits the Met-tRNA_i_. Also, a 48S PIC missing only the Met-tRNA_i_ would be functionally similar to a 48S PIC missing the entire TC. The general notion is that under normal conditions the TC is always present on the scanning 48S PIC and absent only under special conditions. The TC is not required for scanning, as evidenced by the mechanism of translation re-initiation after an uORF [61]. However, it is required for start codon selection – without the Met-tRNA_i_, the 48S PIC cannot discriminate between a start codon and any other codon. Therefore, it must be present on every 48S PIC before it can select a start codon. However, it is possible that, during ISR, when the TC levels are lower, incomplete 43S PICs can be assembled – containing eIF2-GDP, but not Met-tRNA_i_, and/or PICs missing the entire TC. This possibility was suggested, for example, by Sokabe and co-authors, who found that the eIF2 interaction with the MFC is independent of the bound nucleotide, while the MFC strongly discriminates against Met-tRNA_i_ binding to eIF2-GDP [15]. If such a Met-tRNA_i_-less 43S PIC can bind to the mRNA and start scanning, it would enable regulatory mechanisms similar to those mediated by translation re-initiation after an uORF but distinct from them and complementary.

When is eIF2-GDP released from the 48S PIC, and when does eIF5B come? Upon start codon selection and GTP hydrolysis, eIF2-GDP is replaced by eIF5B. But is it replaced or displaced? It has long been known that the eIF2-GDP affinity for Met-tRNA_i_ is not negligible and that eIF2-GDP release may not be instantaneous [62,63]. Furthermore, if there is a long delay between eIF2 release and eIF5B binding, there is a risk of the labile bond between the tRNA and the methionine getting hydrolyzed. Previously, it was reported that eIF2-GDP hangs around on the 48S PIC for a while, some even surviving ultracentrifugation, and that eIF5B promotes its release [63]. Later, we reported that in mammals (but not *S. cerevisiae*), eIF5 binds directly to eIF5B and proposed that eIF5B is recruited by eIF5 before it is needed (before GTP hydrolysis by eIF2) and displaces eIF2-GDP upon GTP hydrolysis [64]. New late-48S PIC structures show that eIF2-GDP does indeed remain on the 48S PIC and is indeed displaced from the Met-tRNA_i_ by eIF5B, and even remains associated with the 48S PIC after it has lost contact with the Met-RNAi. The authors did not observe eIF5B on the 48S PIC until after eIF5-NTD replaced eIF1 on the 40S [19]. Also, if as discussed above, eIF5 itself does indeed arrive only after start codon selection, that is the earliest it can bring eIF5B with itself. Thus, like with eIF5, the ‘eIF5B pendulum’ may have swung back and forth, too – from the original idea that eIF5B comes after eIF2-GDP has left, to eIF5B being present already on the scanning 48S PIC, back to maybe coming before GTP hydrolysis by eIF2 but after start codon selection. If eIF5 does bind dynamically to the 48S PIC before start codon selection, the known interaction between eIF5-CTT and eIF5B-CTD (*Kd* about 500 nM) [64] may not be sufficient to keep eIF5B on the 48S PIC without additional contacts between eIF5B and other PIC components. But since eIF5B is not observed in early-stage PICs and its binding site on the 40S subunit may be occupied by ABCE1 [17], such putative interactions would need to be dynamic as well – perhaps mediated by the mostly disordered N-terminal half of eIF5B? It is intriguing that in the new late-48S PIC structure [19], eIF5B-CTD contacts eIF5-NTD – a novel interaction that has never before been observed.

Another set of outstanding questions relate to interactions observed in solution but not in PIC structures. For example, while it is safe to assume that eIF2β-NTT binds to eIF5-CTD and 5MP-CTD both off and on the PIC (before and/or only after start codon selection), it is not clear when eIF5-CTD binds to eIF1, eIF1A, and eIF3c, when 5MP binds to eIF3c, or whether the timing of these interactions is conserved between mammals and yeast. The interaction between eIF1 and eIF5 is rather weak, mediated by eIF5-CTD, and the respective surface on eIF1 is occupied by other interactions when it is bound to the PIC; therefore, eIF1 and eIF5 probably only contact each other within the MFC off the ribosome. However, the interactions of eIF5-CTD and 5MP-CTD with eIF3c were reported to be important for the stringency of start codon selection and thus must be happening on the 48S PIC (but remain invisible in structures; thus, must be tethered via flexible linkers). Likewise, eIF1A has not been reported to be part of the MFC. Therefore, it would likely be binding eIF5 within the PIC. The main interaction involves the N-terminus of eIF1A; thus, it would be possible at stages, where the N-terminus of eIF1A remains disordered within the PIC (otherwise, eIF5-CTD would have been observed bound to eIF1A in the PIC structure). It is even harder to guess when the eIF5-CTD interaction with the folded domain of eIF1A could occur. Finally, while a single helix from eIF3c is seen in PIC structures contacting eIF1 [16–20], eIF3c appears to have multiple eIF1- and eIF5-binding sites in *S. cerevisiae* [21]. The location of the eIF1-binding region in mammalian eIF3c is different than in *S. cerevisiae*, while the sequences appear similar, and this difference could be due to translocation, as opposed to convergent evolution [16,17]. Thus, it remains to be elucidated how many eIF1- or eIF5-binding sites exist in eIF3c from different kingdoms of life and which interactions can occur on the PIC, fully or partially.

In summary, it is now well established that many more interactions within the PIC are dynamic and that the PICs themselves are more heterogeneous than previously thought. In fact, we may be better off assuming that the association of a given eIF with the PIC may be dynamic until proven otherwise. Figure 2 attempts to illustrate most points in translation initiation at which each of the proteins discussed here binds to the PIC or dissociates from it. This emphasizes the need for quantitative approaches to better understand the process of translation initiation. In this context, kinetics experiments measuring the lifetimes of individual complexes are invaluable. Another quantitative approach with great potential is considering the concentrations of ribosomes and eIFs in different cells and under different conditions. Unfortunately, the cellular concentrations of proteins and other molecules are notoriously difficult to measure accurately, and the available numbers are contradictory in proteomics databases and even in studies specifically focused on ribosomes and translation factors, varying by up to an order of magnitude [65–67]. Although some recent studies report more realistic numbers and ratios of translation factors to ribosomes [66,68].

**Figure 2:**
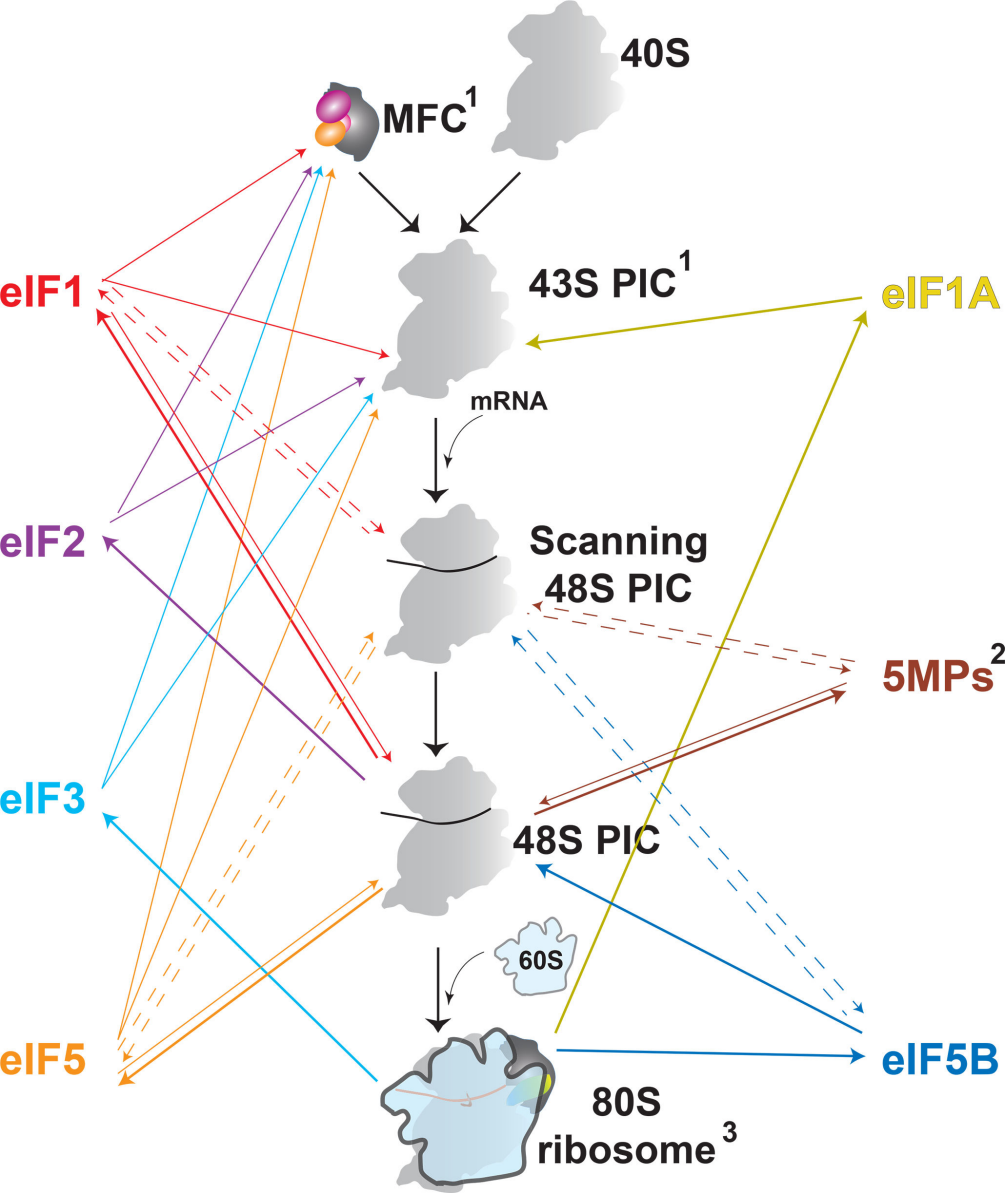
Timing and dynamics of eIF binding and release within the pre-initiation complex (PIC). The diagram only includes eIF2 and the other proteins discussed in this review. Other proteins, such as the cap-binding complex eIF4F (eIF4E/G/A), are not shown. Not all shown interactions may take place in both mammals and *S. cerevisiae*. Protein binding and release are shown with color-coded arrows pointing away from, or toward the protein, respectively. Bold lines indicate the main (or only) binding/release point in the process. Bi-directional arrows indicate dynamic reversible binding. Dashed arrows indicate putative interactions. Notes: 1. MFC components may bind to the 43S PIC as a pre-formed MFC or individually directly to the 43S PIC. Binding within the MFC, and even the 43S PIC may be dynamic, at least for some of the proteins; although, this would be unlikely to have an effect on the process. 2. The 5MPs could also dynamically bind to the MFC and the 43S PIC and compete with eIF5 (not shown). 3. eIF5B is released from the 80S PIC, whereas eIF3 is released from the translating ribosome, along with eIF4F (not shown). eIFs 1 and 1A have binding sites on eIF3 and could also bind dynamically to eIF3 on translating ribosomes (not shown), which would help them show up on time for translation reinitiation, after the ribosome has translated a short uORF. 40S, small ribosomal subunit. 60S, large ribosomal subunit.

Another factor to consider is the presence of membrane-less protein condensates, which appear to serve a wide range of functions, including sequestering mRNAs and translation apparatus components, degradation, and translation factories, where certain mRNAs and translation complexes are concentrated (reviewed in [69,70]). Protein condensates are often nucleated by intrinsically disordered regions (IDRs) in proteins. A number of eIFs contain extensive IDRs, including eIF3 subunits, eIF4G, and eIF5B, some of which have already been reported to form protein condensates involved in regulation of translation (see e.g., [71,72]). Such condensates pose potential challenges because they make it difficult to estimate the actual local mRNA and protein concentrations at the site, where translation takes place but also open exciting new avenues for research.

Combining PIC structures, ribosome and eIF concentrations, and SM-FRET and other kinetics data will allow a better mechanistic understanding of the process by performing an ‘accounting’ of the translation apparatus – how many molecules of a given component are there, who are they bound to, for how long, what are they doing, and how long does every step of the process take on average?

**Perspectives**

- Eukaryotic translation initiation factor 2 (eIF2) is a trimeric GTPase at the center of the translation initiation process and its regulation. GTP hydrolysis by eIF2, stimulated by eIF5, serves as a checkpoint during start codon selection, committing the scanning 48S pre-initiation complex (PIC) to initiate at the current start codon, while phosphorylation of eIF2 by any of the stress-induced kinases triggers the integrated stress response.
- Recently reported PIC structures show where and when several key interactions take place, while single-molecule experiments emphasize the dynamic nature of some of these interactions in the context of the PIC. These advances help elucidate the mechanisms of translation initiation, and especially the stringency of start codon selection.
- Future progress in elucidating translation initiation would be helped greatly by combining structures of PICs and other translation complexes, kinetics analysis, for example, by SM-FRET, and analysis of ribosome and translation factor abundances, in order to obtain a detailed quantitative ‘accounting’ of all the components of the translational apparatus at every stage of the process.

## Abbreviations

GAP: GTPase-activating protein
GEF: guanine nucleotide exchange factor
ISR: integrated stress response
MFC: multifactor complex
PIC: pre-initiation complex
TC: ternary complex
eIF2: eukaryotic translation initiation factor 2
uORF: upstream open reading frame

## References

[1] Hinnebusch A.G (2014). The scanning mechanism of eukaryotic translation initiation. Annu. Rev. Biochem..

[2] Hershey J.W.B., Sonenberg N., Mathews M.B (2019). Principles of translational control. Cold Spring Harb. Perspect. Biol..

[3] Dever T.E., Ivanov I.P., Hinnebusch A.G (2023). Translational regulation by uORFs and start codon selection stringency. Genes Dev..

[4] Brito Querido J., Díaz-López I., Ramakrishnan V (2024). The molecular basis of translation initiation and its regulation in eukaryotes. Nat. Rev. Mol. Cell Biol..

[5] Jackson R.J., Hellen C.U.T., Pestova T.V (2010). The mechanism of eukaryotic translation initiation and principles of its regulation. Nat. Rev. Mol. Cell Biol..

[6] Bohlen J., Fenzl K., Kramer G., Bukau B., Teleman A.A (2020). Selective 40S footprinting reveals cap-tethered ribosome scanning in human cells. Mol. Cell.

[7] Gu Y., Mao Y., Jia L., Dong L., Qian S.B (2021). Bi-directional ribosome scanning controls the stringency of start codon selection. Nat. Commun..

[8] Kumar P., Hellen C.U.T., Pestova T.V (2016). Toward the mechanism of eIF4F-mediated ribosomal attachment to mammalian capped mRNAs. Genes Dev..

[9] Archer S.K., Shirokikh N.E., Beilharz T.H., Preiss T (2016). Dynamics of ribosome scanning and recycling revealed by translation complex profiling. Nature.

[10] Berthelot K., Muldoon M., Rajkowitsch L., Hughes J., McCarthy J.E.G (2004). Dynamics and processivity of 40S ribosome scanning on mRNA in yeast. Mol. Microbiol..

[11] Lin Y., Li F., Huang L., Polte C., Duan H., Fang J. (2020). eIF3 associates with 80S ribosomes to promote translation elongation, mitochondrial homeostasis, and muscle health. Mol. Cell.

[12] Pöyry T.A.A., Kaminski A., Jackson R.J (2004). What determines whether mammalian ribosomes resume scanning after translation of a short upstream open reading frame?. Genes Dev..

[13] Marintchev A., Ito T (2020). eIF2B and the integrated stress response: a structural and mechanistic view. Biochemistry.

[14] Asano K., Clayton J., Shalev A., Hinnebusch A.G (2000). A multifactor complex of eukaryotic initiation factors, eIF1, eIF2, eIF3, eIF5, and initiator tRNA(Met) is an important translation initiation intermediate *in vivo*. Genes Dev..

[15] Sokabe M., Fraser C.S., Hershey J.W.B (2012). The human translation initiation multi-factor complex promotes methionyl-tRNAi binding to the 40S ribosomal subunit. Nucleic Acids Res..

[16] Brito Querido J., Sokabe M., Kraatz S., Gordiyenko Y., Skehel J.M., Fraser C.S. (2020). Structure of a human 48*S* translational initiation complex. Science.

[17] Kratzat H., Mackens-Kiani T., Ameismeier M., Potocnjak M., Cheng J., Dacheux E. (2021). A structural inventory of native ribosomal ABCE1-43S pre-initiation complexes. EMBO J..

[18] Llácer J.L., Hussain T., Marler L., Aitken C.E., Thakur A., Lorsch J.R. (2015). Conformational differences between open and closed states of the eukaryotic translation initiation complex. Mol. Cell.

[19] Petrychenko V., Yi S.H., Liedtke D., Peng B.Z., Rodnina M.V., Fischer N (2025). Structural basis for translational control by the human 48S initiation complex. Nat. Struct. Mol. Biol..

[20] Yi S.H., Petrychenko V., Schliep J.E., Goyal A., Linden A., Chari A. (2022). Conformational rearrangements upon start codon recognition in human 48S translation initiation complex. Nucleic Acids Res..

[21] Obayashi E., Luna R.E., Nagata T., Martin-Marcos P., Hiraishi H., Singh C.R. (2017). Molecular landscape of the ribosome pre-initiation complex during mRNA scanning: structural role for eIF3c and its control by eIF5. Cell Rep..

[22] Asano K., Krishnamoorthy T., Phan L., Pavitt G.D., Hinnebusch A.G (1999). Conserved bipartite motifs in yeast eIF5 and eIF2Bepsilon, GTPase-activating and GDP-GTP exchange factors in translation initiation, mediate binding to their common substrate eIF2. EMBO J..

[23] Das S., Maiti T., Das K., Maitra U (1997). Specific interaction of eukaryotic translation initiation factor 5 (eIF5) with the beta-subunit of eIF2. J. Biol. Chem..

[24] Luna R.E., Arthanari H., Hiraishi H., Akabayov B., Tang L., Cox C. (2013). The interaction between eukaryotic initiation factor 1A and eIF5 retains eIF1 within scanning preinitiation complexes. Biochemistry.

[25] Luna R.E., Arthanari H., Hiraishi H., Nanda J., Martin-Marcos P., Markus M.A. (2012). The C-terminal domain of eukaryotic initiation factor 5 promotes start codon recognition by its dynamic interplay with eIF1 and eIF2β. Cell Rep..

[26] Yamamoto Y., Singh C.R., Marintchev A., Hall N.S., Hannig E.M., Wagner G. (2005). The eukaryotic initiation factor (eIF) 5 HEAT domain mediates multifactor assembly and scanning with distinct interfaces to eIF1, eIF2, eIF3, and eIF4G. Proc. Natl. Acad. Sci. U.S.A..

[27] Gamble N., Paul E.E., Anand B., Marintchev A (2022). Regulation of the interactions between human eIF5 and eIF1A by the CK2 kinase. Curr. Res. Struct. Biol..

[28] Bochler A., Querido J.B., Prilepskaja T., Soufari H., Simonetti A., Del Cistia M.L. (2020). Structural differences in translation initiation between pathogenic trypanosomatids and their mammalian hosts. Cell Rep..

[29] Ivanov I.P., Loughran G., Sachs M.S., Atkins J.F (2010). Initiation context modulates autoregulation of eukaryotic translation initiation factor 1 (eIF1). Proc. Natl. Acad. Sci. U.S.A..

[30] Loughran G., Sachs M.S., Atkins J.F., Ivanov I.P (2012). Stringency of start codon selection modulates autoregulation of translation initiation factor eIF5. Nucleic Acids Res..

[31] Martin-Marcos P., Cheung Y.N., Hinnebusch A.G (2011). Functional elements in initiation factors 1, 1A, and 2β discriminate against poor AUG context and non-AUG start codons. Mol. Cell. Biol..

[32] Kozel C., Thompson B., Hustak S., Moore C., Nakashima A., Singh C.R. (2016). Overexpression of eIF5 or its protein mimic 5MP perturbs eIF2 function and induces ATF4 translation through delayed re-initiation. Nucleic Acids Res..

[33] Singh C.R., Glineburg M.R., Moore C., Tani N., Jaiswal R., Zou Y. (2021). Human oncoprotein 5MP suppresses general and repeat-associated non-AUG translation via eIF3 by a common mechanism. Cell Rep..

[34] Tang L., Morris J., Wan J., Moore C., Fujita Y., Gillaspie S. (2017). Competition between translation initiation factor eIF5 and its mimic protein 5MP determines non-AUG initiation rate genome-wide. Nucleic Acids Res..

[35] Marintchev A., Wagner G (2005). eIF4G and CBP80 share a common origin and similar domain organization: implications for the structure and function of eIF4G. Biochemistry.

[36] Jumper J., Evans R., Pritzel A., Green T., Figurnov M., Ronneberger O. (2021). Highly accurate protein structure prediction with AlphaFold. Nature.

[37] Varadi M., Anyango S., Deshpande M., Nair S., Natassia C., Yordanova G. (2022). AlphaFold protein structure database: massively expanding the structural coverage of protein-sequence space with high-accuracy models. Nucleic Acids Res..

[38] Koonin E.V (1995). Multidomain organization of eukaryotic guanine nucleotide exchange translation initiation factor eIF-2B subunits revealed by analysis of conserved sequence motifs. Protein Sci..

[39] Loughran G., Firth A.E., Atkins J.F., Ivanov I.P (2018). Translational autoregulation of BZW1 and BZW2 expression by modulating the stringency of start codon selection. Plos One.

[40] Asano K (2021). Origin of translational control by eIF2α phosphorylation: insights from genome-wide translational profiling studies in fission yeast. Curr. Genet..

[41] Hiraishi H., Oatman J., Haller S.L., Blunk L., McGivern B., Morris J. (2014). Essential role of eIF5-mimic protein in animal development is linked to control of ATF4 expression. Nucleic Acids Res..

[42] Algire M.A., Maag D., Lorsch J.R (2005). Pi release from eIF2, not GTP hydrolysis, is the step controlled by start-site selection during eukaryotic translation initiation. Mol. Cell.

[43] Singh C.R., Lee B., Udagawa T., Mohammad-Qureshi S.S., Yamamoto Y., Pavitt G.D. (2006). An eIF5/eIF2 complex antagonizes guanine nucleotide exchange by eIF2B during translation initiation. EMBO J..

[44] Jennings M.D., Pavitt G.D (2010). eIF5 has GDI activity necessary for translational control by eIF2 phosphorylation. Nature.

[45] Jennings M.D., Zhou Y., Mohammad-Qureshi S.S., Bennett D., Pavitt G.D (2013). eIF2B promotes eIF5 dissociation from eIF2*GDP to facilitate guanine nucleotide exchange for translation initiation. Genes Dev..

[46] DerMardirossian C., Bokoch G.M (2005). GDIs: central regulatory molecules in Rho GTPase activation. Trends Cell Biol..

[47] Wagner P.A., Song M., Ficner R., Kuhle B., Marintchev A (2024). Molecular basis for the interactions of eIF2β with eIF5, eIF2B, and 5MP1 and their regulation by CK2. bioRxiv.

[48] Gandin V., Masvidal L., Cargnello M., Gyenis L., McLaughlan S., Cai Y. (2016). mTORC1 and CK2 coordinate ternary and eIF4F complex assembly. Nat. Commun..

[49] Homma M.K., Wada I., Suzuki T., Yamaki J., Krebs E.G., Homma Y (2005). CK2 phosphorylation of eukaryotic translation initiation factor 5 potentiates cell cycle progression. Proc. Natl. Acad. Sci. U.S.A..

[50] Paytubi S., Morrice N.A., Boudeau J., Proud C.G (2008). The N-terminal region of ABC50 interacts with eukaryotic initiation factor eIF2 and is a target for regulatory phosphorylation by CK2. Biochem. J..

[51] Turowec J.P., Duncan J.S., French A.C., Gyenis L., Denis N.A., Vilk G. (2010). Protein kinase CK2 is a constitutively active enzyme that promotes cell survival: strategies to identify CK2 substrates and manipulate its activity in mammalian cells. Meth. Enzymol..

[52] Lamper A.M., Fleming R.H., Ladd K.M., Lee A.S.Y (2020). A phosphorylation-regulated eIF3d translation switch mediates cellular adaptation to metabolic stress. Science.

[53] Lee A.S., Kranzusch P.J., Doudna J.A., Cate J.H.D (2016). eIF3d is an mRNA cap-binding protein that is required for specialized translation initiation. Nature.

[54] Mukhopadhyay S., Amodeo M.E., Lee A.S.Y (2023). eIF3d controls the persistent integrated stress response. Mol. Cell.

[55] Singh L.P., Denslow N.D., Wahba A.J (1996). The interaction of rabbit reticulocyte guanine nucleotide exchange factor eIF-2B with chain initiation factor 2: studies with N-ethylmaleimide and trypsin. Biochem. Biophys. Res. Commun..

[56] Paul E.E., Lin K.Y., Gamble N., Tsai A.W.L., Swan S.H.K., Yang Y. (2022). Dynamic interaction network involving the conserved intrinsically disordered regions in human eIF5. Biophys. Chem..

[57] Pisareva V.P., Pisarev A.V (2014). eIF5 and eIF5B together stimulate 48S initiation complex formation during ribosomal scanning. Nucleic Acids Res..

[58] Terenin I.M., Akulich K.A., Andreev D.E., Polyanskaya S.A., Shatsky I.N., Dmitriev S.E (2016). Sliding of a 43S ribosomal complex from the recognized AUG codon triggered by a delay in eIF2-bound GTP hydrolysis. Nucleic Acids Res..

[59] Llácer J.L., Hussain T., Saini A.K., Nanda J.S., Kaur S., Gordiyenko Y. (2018). Translational initiation factor eIF5 replaces eIF1 on the 40S ribosomal subunit to promote start-codon recognition. Elife.

[60] Grosely R., Alvarado C., Ivanov I.P., Nicholson O.B., Puglisi J.D., Dever T.E. (2024). eIF1 and eIF5 dynamically control translation start site fidelity. bioRxiv.

[61] Hinnebusch A.G., Jackson B.M., Mueller P.P (1988). Evidence for regulation of reinitiation in translational control of GCN4 mRNA. Proc. Natl. Acad. Sci. U.S.A..

[62] Kapp L.D., Lorsch J.R (2004). GTP-dependent recognition of the methionine moiety on initiator tRNA by translation factor eIF2. J. Mol. Biol..

[63] Pisarev A.V., Kolupaeva V.G., Pisareva V.P., Merrick W.C., Hellen C.U.T., Pestova T.V (2006). Specific functional interactions of nucleotides at key -3 and +4 positions flanking the initiation codon with components of the mammalian 48S translation initiation complex. Genes Dev..

[64] Lin K.Y., Nag N., Pestova T.V., Marintchev A (2018). Human eIF5 and eIF1A compete for binding to eIF5B. Biochemistry.

[65] Duncan R., Hershey J.W (1983). Identification and quantitation of levels of protein synthesis initiation factors in crude HeLa cell lysates by two-dimensional polyacrylamide gel electrophoresis. J. Biol. Chem.

[66] Singh C.R., Udagawa T., Lee B., Wassink S., He H., Yamamoto Y. (2007). Change in nutritional status modulates the abundance of critical pre-initiation intermediate complexes during translation initiation *in vivo*. J. Mol. Biol..

[67] Von der Haar T., McCarthy J.E.G (2002). Intracellular translation initiation factor levels in Saccharomyces cerevisiae and their role in cap-complex function. Mol. Microbiol..

[68] Schwanhäusser B., Busse D., Li N., Dittmar G., Schuchhardt J., Wolf J. (2011). Global quantification of mammalian gene expression control. Nature.

[69] Nguyen D.T.M., Koppers M., Farías G.G (2024). Endoplasmic reticulum - condensate interactions in protein synthesis and secretion. Curr. Opin. Cell Biol..

[70] Parker D.M., Winkenbach L.P., Osborne Nishimura E (2022). It’s just a phase: exploring the relationship between mRNA, biomolecular condensates, and translational control. Front. Genet..

[71] Harris M.T., Marr M.T (2023). The intrinsically disordered region of eIF5B stimulates IRES usage and nucleates biological granule formation. Cell Rep..

[72] Iserman C., Desroches Altamirano C., Jegers C., Friedrich U., Zarin T., Fritsch A.W. (2020). Condensation of Ded1p promotes a translational switch from housekeeping to stress protein production. Cell.

